# Genome-wide linkage and exome analyses identify variants of *HMCN1* for splenic epidermoid cyst

**DOI:** 10.1186/s12881-014-0115-4

**Published:** 2014-10-23

**Authors:** Waleed H Omer, Akira Narita, Kazuyoshi Hosomichi, Shigeki Mitsunaga, Yasuhiro Hayashi, Atsushi Yamashita, Avdyl Krasniqi, Yuri Iwasaki, Masami Kimura, Ituro Inoue

**Affiliations:** Division of Human Genetics, National Institute of Genetics, The Graduate University for Advanced Studies (SOKENDAI), Yata 1111, Mishima, 411-8540 Shizuoka Japan; Department of Molecular Life Sciences, Basic Medical Science and Molecular Medicine, Tokai University School of Medicine, Shimokasuya143, Isehara, 259-1193 Kanagawa Japan; Faculty of Pharma-Sciences, Teikyo University, Kaga, 2-11-1, Itabashi-ku, Tokyo, 173-8605 Japan; Department of Abdominal Surgery, University Clinical Center of Kosovo, 10 000 Prishtina, Kosovo; Health Insurance Hitoyoshi General Hospital, Oikami-machi 35, Hitoyoshi, 868-8555 Kumamoto Japan; Present address: StaGen Co., Ltd., KUGA Building 8F, 4-11-6 Kuramae, Taito-ku, Tokyo, 111-0051 Japan

**Keywords:** Exome re-sequencing, Splenic epidermoid cyst, *HMCN1*, Linkage analysis

## Abstract

**Background:**

Splenic epidermoid cyst is a benign tumor-like lesion affecting the spleen and sometimes occurs in familial form. The causality of such rare diseases remain challenging, however recently, with the emergence of exome re-sequencing, the genetics of many diseases have been unveiled. In the present study, we performed a combinatorial approach of genome-wide parametric linkage and exome analyses for a moderate-sized Japanese family with frequent occurrence of splenic epidermoid cyst to identify the genetic causality of the disease.

**Methods:**

Twelve individuals from the family were subject to SNP typing and exome re-sequencing was done for 8 family members and 4 unrelated patients from Kosovo. Linkage was estimated using multi-point parametric linkage analysis assuming a dominant mode of inheritance. All of the candidate variants from exome analysis were confirmed by direct sequencing.

**Results:**

The parametric linkage analysis suggested two loci on 1q and 14q with a maximal LOD score of 2.5 . Exome generated variants were prioritized based on; impact on the protein coding sequence, novelty or rareness in public databases, and position within the linkage loci. This approach identified three variants; variants of *HMCN1* and *CNTN2* on 1q and a variant of *DDHD1* on 14q. The variant of *HMCN1* (p.R5205H) showed the best co-segregation in the family after validation with Sanger sequencing. Additionally, rare missense variants (p.A4704V, p.T5004I, and p.H5244Q) were detected in three unrelated Kosovo patients. The identified variants of *HMCN1* are on conserved domains, particularly the two variants on calcium-binding epidermal growth factor domain.

**Conclusions:**

The present study, by combining linkage and exome analyses, identified *HMCN1* as a genetic causality of splenic epidermoid cyst. Understanding the biology of the disease is a key step toward developing innovative approaches of intervention.

**Electronic supplementary material:**

The online version of this article (doi:10.1186/s12881-014-0115-4) contains supplementary material, which is available to authorized users.

## Background

Mendelian disorders are classically analyzed by linkage analysis to determine genetic loci and subsequently positional cloning to identify the causal variants. With the advent of deep sequencing technologies, re-sequencing of entire human genome could become a powerful tool to identify variants underlying monogenic and probably common disorders. Although protein-coding regions constitute approximately 1% of the human genome, 85% of the variants with substantial effects on disease-related traits locate on those regions [1]. Accordingly, exome re-sequencing has increasingly been used as an efficient way to identify genetic causes of diseases [2,3]. However, exome re-sequencing alone would still leave a considerable number of candidate variants. In the current study, we performed exome re-sequencing, in samples from a Japanese family and 4 unrelated Kosovar having splenic epidermoid cyst, in combination with genome-wide parametric linkage analysis as filtering procedure, to identify the genetic causality [4].

**Figure 1:**
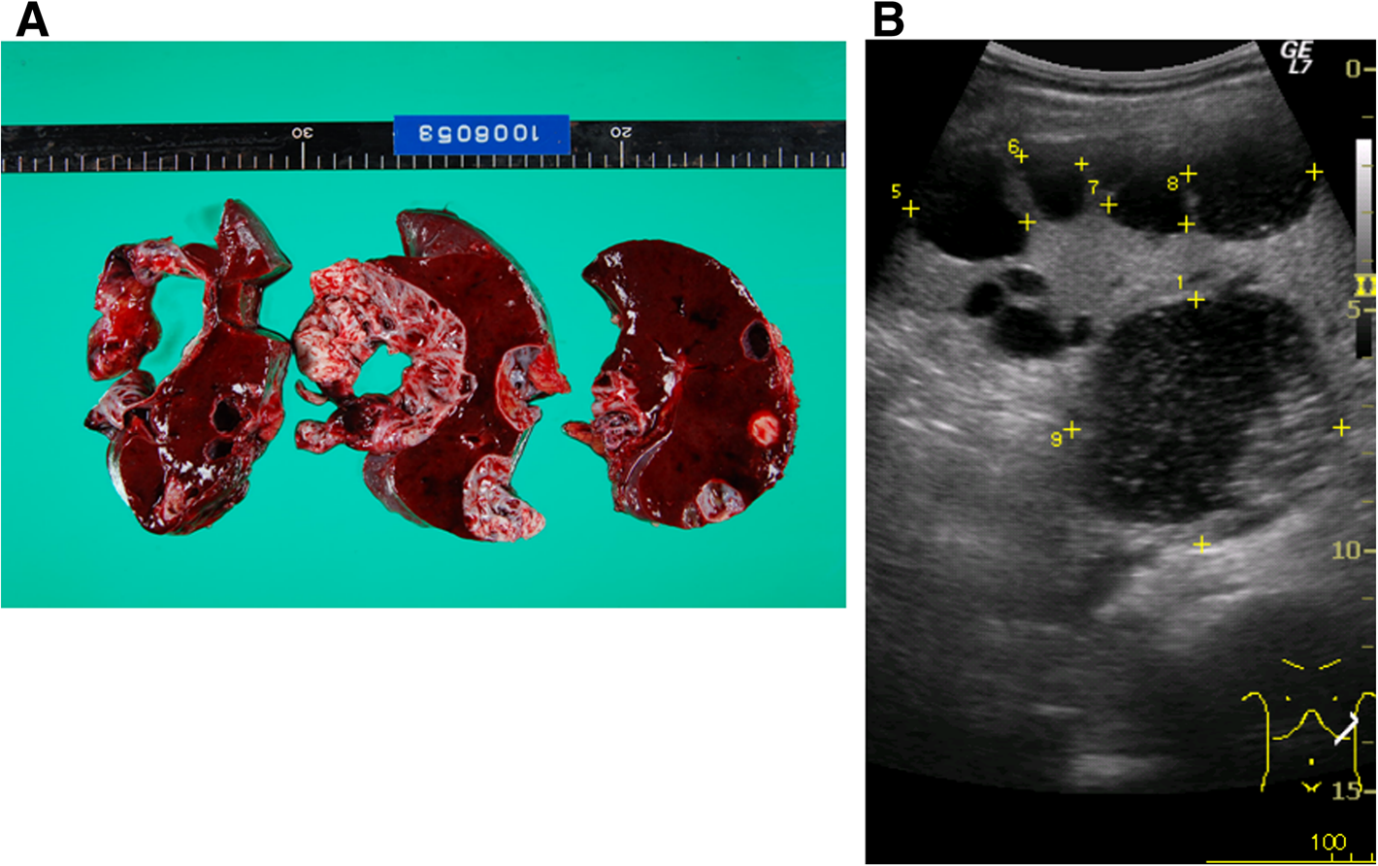
**Clinical features of splenic epidermoid cyst. (A)** Photograph of the surgical specimen from the spleen of the proband (IV:3) of the Japanese family. **(B)** Abdominal ultrasound image showing the splenic cysts of IV:3. Multiple tumor-like lesions were seen filled with liquid or semi-liquid material and pathological examination showed that the walls of the cysts consisted of vitrificated fibrous connective tissues, lined by stratified squamous epithelia.

Splenic epidermoid cyst is a very rare benign tumor-like lesion filled with fluid or semi-fluid material (Figure 1). It occurs mostly at the 2^nd^ or 3^rd^ decade of life with a female dominance [5]. Most cases are asymptomatic and the clinical symptoms are dependent on the size of the cyst. Cysts larger than 5 cm, as well as symptomatic and complicated cysts that may cause hemorrhage, rupture, or infection, should be surgically treated [6,7]. While cysts are usually singular, multiple cysts have been observed especially in familial cases. Splenic cysts are much less common than those arising in kidney, liver, or ovary [8], and the molecular etiology of this condition is mostly unknown. A possible etiology of the cyst formation is attributed to developmental stages when misplacement of epithelial tissue occurs and also to metaplasia [9], however, the developmental origin of the epidermoid cysts of the spleen is unclear. Thus far, several affected parent–child and sibs have been reported [9-14], suggesting that genetic factors are possibly involved in predisposition to the disease. In the current study, in order to decipher the genetic factor, we recruited a Japanese family with frequent occurrences of splenic epidermoid cyst and performed genome-wide linkage analysis and exome capture followed by massive parallel sequencing. A series of filtering steps identified three candidate genes and then patients recruited in Kosovo were used for replication to finally identify a single candidate gene.

## Methods

### Study subjects

A three-generation Japanese family comprising seventeen members (five males/twelve females), including six patients of splenic epidermoid cyst, was recruited at Health Insurance Hitoyoshi General Hospital, Kumamoto Prefecture, Japan. The proband (IV:3), a 16-year-old girl, exhibited abdominal pain during exercise and later diagnosed as having multiple epidermoid cysts in spleen using ultrasonography and CT scan (Figure 1). Laboratory data of the patient’s serum showed elevated level of carbohydrate antigen 19–9 (CA19-9) i.e. 329 units/ml (<37 units/ml), which returned to the normal range after splenectomy. Since the proband’s father (III:2) was diagnosed with the disease and treated with splenectomy, we started extending recruitment to the other family members. Recruited members were examined for splenic cysts by ultrasound or at least clinical examination of the spleen in case of ultrasound was not possible. In addition to this Japanese family, we ascertained four DNA samples of unrelated affected individuals from Kosovo. The patients underwent surgery and their final diagnosis was pathologically confirmed with the resected specimens. Considering the rarity of splenic cyst, increasing the sample size, particularly from a different population, would strengthen the significance of the findings. The study was approved by the ethical committees of the Health Insurance Hitoyoshi General Hospital, University Clinical Center of Kosovo, Prishtina University, School of Medicine, and the Tokai University, School of Medicine. All of the participants or their guardians gave written informed consent.

### SNP genotyping and linkage analysis

**Figure 2:**
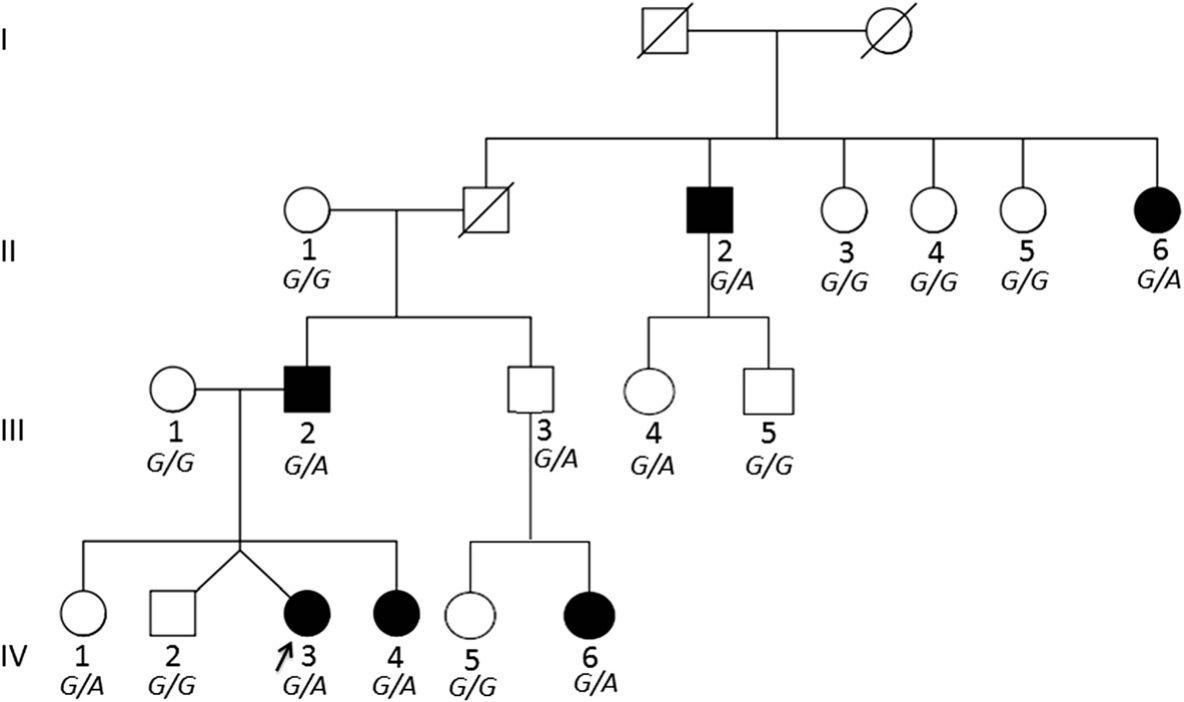
**Pedigree of a Japanese family with splenic epidermoid cyst and co-segregation of R5205H variant of** ***HMCN1*** **.** Filled squares and circles denote affected individuals and open symbols represent unaffected subjects. The arrow indicates the proband (IV:3). The genotypes are for the R5205H variant of *HMCN1*, which is co-segregating with the affection status except for III-3, III-4, and IV-1.

Genomic DNA was extracted from peripheral blood or saliva samples according to the standard protocol. The DNA samples (250 ng) from 12 family members were subjected to genotyping using the Genome-Wide Human SNP Array 5.0 (Affymetrix, Santa Clara, CA, USA) following the manufacturer’s recommendations. The 12 member were II:1, II:2, II:4, II:6, III:1, III:2, III:3, IV:2, IV:3, IV:4, IV:5, and IV:6 (Figure 2). III:3 is an unaffected individual but also the father of the affected child (IV:6). We assumed III:3 is a carrier of the causal variant but we assigned him as unaffected in the linkage analysis. A total of 443,816 single nucleotide polymorphisms (SNPs) were genotyped. Then monomorphic or X-linked SNPs and SNPs showing Mendelian inconsistencies were excluded; leaving 274,743 SNPs for further analysis. However there were some concerns about performing linkage with such number of SNPs (i.e. inflation of linkage statistics due to linkage disequilibrium [15], effect of typing errors, and impractical use of memory). To circumvent these difficulties, we divided the data into 30 subsets by taking one SNP every 30 successive SNPs; Thus, around 9,200 SNPs in each dataset at interval of approximately 0.34 Mb (see Additional file 1). We then performed the linkage analysis on each of the 30 subsets, and calculated the average LOD scores of all the subsets. Looking at the affection status in the family’s pedigree indicated that the dominant mode of inheritance was most conceivable. We performed multi-point parametric linkage analysis using MERLIN 1.1.2 [16] with penetrance levels setting as follows: under a dominant mode, 0.95 for *Aa* and *aa* genotypes (*A* and *a* denote wild-type and mutant alleles, respectively) and 0.005 for *AA* genotype. Considering rarity of the disease, frequency of disease-causing allele was set to 5.0 × 10^−3^.

### Exome re-sequencing and data analysis

For exome re-sequencing, we selected eight individuals form the Japanese family (II:1, II:2, II:3, II:5, III:1, III:2, IV:3, and IV:4), which included four affected members. In addition, exome re-sequencing was done for four unrelated affected individuals from Kosovo. Starting with 3 μg of the DNA, samples were subject to exome capture using Sure-Select Human All Exon Kit (Agilent Technologies, Santa Clara, CA, USA), according to the manufacturer’s procedures. The captured DNA was sequenced using HiSeq 2000 (illumina, San Diego, CA, USA). The reads were mapped to the reference genome (UCSC hg19) using BWA v.0.5.7 [17]. BWA generated SAM files were sorted and indexed using SAMtools v.0.1.7a [18] then duplicated reads were marked with Picard v.1.102 [19]. The files obtained in BAM format were analyzed using GATK v.2.7 following their best practices guidelines [20]. In brief, the BAM files were first subject to indel realignment, base quality score recalibration, and then variants calling with the Unified Genotyper walker, to obtain the potential variants in a VCF file. Variants in VCF files were annotated using the algorithm (table.annovar.pl) in ANNOVAR (version 2013jul21) [21]. For annotation of genes, we used RefSeq gene database (build hg19) [22], while variants’ annotation was based on dbSNP (dbSNP 137) and 1000 Genomes Project databases [23].

### Sanger sequencing

We examined all the family members by Sanger sequencing to confirm the finding as well as co-segregation of the variants with the disease status. PCR primers, flanking approximately 300 bp of the final candidate variants, were designed using Primer3Plus [24] and the target sequences were amplified by PCR then sequenced using Sanger method on the 3730 DNA Analyzer (Applied Biosystems, Tokyo, Japan).

## Results

### Genome-wide linkage analysis

**Figure 3:**
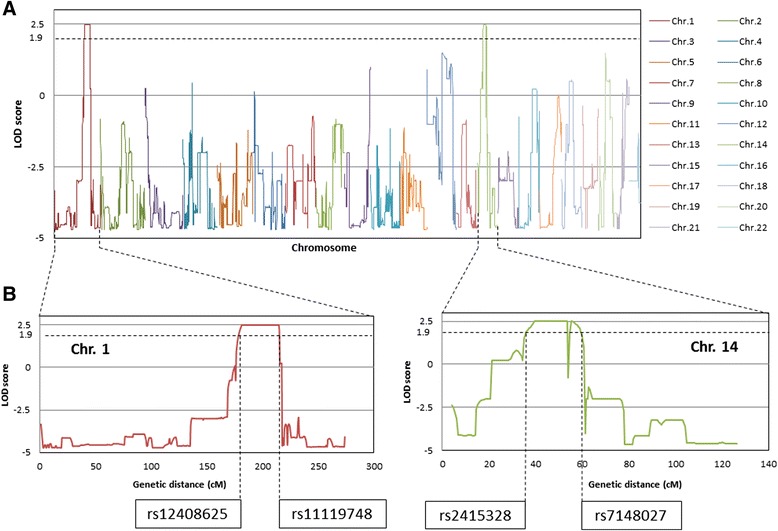
**Plots of SNP-based genome-wide linkage analyses. (A)** LOD scores from the genome-wide multi-point parametric linkage analysis. The horizontal dotted line indicates the threshold LOD = 1.90 which was used in linkage analysis. **(B)** Enlarged plots of the average LOD scores of the two chromosomes 1 and 14, in which suggestive evidence of linkage was observed. A maximal LOD score of 2.50 was observed at 1q (rs12408625-rs11119748; 181,329,186-211,652,185 bp) and 14q (rs2415328-rs7148027; 36,888,532-56953253 bp).

The average LOD scores of the parametric genome-wide linkage analysis are presented in Figure 3A. Based on a dominant model, a linkage signal with maximal LOD score of 2.50 was found on the chromosomal regions 1q (rs12408625-rs11119748; 181,329,186-211,652,185 bp) and 14q (rs2415328-rs7148027; 36,888,532-56,953,253 bp) (Figure 3B). Although the LOD scores did not reach the widely accepted significant LOD score of 3, the LOD score of 2.5 is assumed to be suggestive evidence of linkage. The loci identified on chromosomes 1 and 14 were then used in exome analysis as a filtering criterion for candidate variants identification.

### Exome analysis

We performed exome analysis for a total of 12 individuals; 8 Japanese family members and 4 unrelated cases from Kosovo. After mapping to the reference genome, approximately 7 Gb of sequences were obtained, which have a 93% coverage of all exons with depth of at least 20 reads and seemed to be sufficient for further analyses. After annotation of variants, we performed sequential filtering steps based on the following criteria; first, based on the variant’s impact on the protein coding sequence; it is likely that the causal variant is an amino acid substitution, frame-shift mutation, or splicing site alteration, second, because familial splenic epidermoid cyst is a very rare disease, it is likely that the causal variant either has a low population frequency or not registered in public databases. Considering the previous criteria, we applied two separate filters; one for the novel variants that have not been reported previously in dbSNP (dbSNP 137) or 1000 Genomes databases, while the second one for variants with minor allele frequency (MAF) of up to 2% in the Japanese or European population according to 1000 Genomes database. Finally, we utilized the linkage data by selecting only variants within the previously identified loci on chromosomes 1 (181,329,186-211,652,185 bp) and 14 (36,888,532-56,953,253 bp). This approach of combining linkage and exome analyses has been useful in reducing the number of investigated variants [25]. Furthermore, only variants that are consistently co-segregating with the disease (i.e. shared by all the cases and absent in the unaffected ones) were selected as candidates. Ultimately, three variants were identified; two variants on chromosome 1 and one variant on chromosome 14 (Table 1).

**Table 1:** **Number of variants obtained using different filters in a family of splenic epidermoid cyst**

**Criteria for variants filtering**	**AFFECTED**				**NOT AFFECTED**		UNCONFIRMED*	
	**III:2**	**IV:3**	**IV:4**	**II:2**	**III:1**	**II:1**	**II:3**	**II:5**
**Total (Exonic, Splicing)**	22903	23110	22925	22686	22705	23389	22900	22748
**SNVs (NS, SC)**	10735	10836	10679	10576	10641	10939	10630	10576
**Not in dbSNP or 1000 Genomes**	630	625	606	606	576	687	648	664
**Within linkage region**								
**Chr. 1: 181,329,185-211,652,186 bp**	8	6	8	9	8	6	7	6
**Chr. 14: 36,888,531-58,397,917 bp**	5	4	2	2	3	3	4	2
**Shared by all AFFECTED**	2 (*CNTN2 & DDHD1*)	2	2	2	-	-	1 (*DDHD1)*	1
**INDELs (FS, non-FS)**	218	222	224	232	231	226	232	235
**Not in dbSNP or 1000 Genomes**	23	25	26	23	19	31	32	25
**Within linkage region**								
**Chr. 1: 181,329,185-211,652,186**	0	0	0	0	0	1	0	0
**Chr. 14: 36,888,531-58,397,917**	0	0	0	0	0	0	0	0
**Rare variants**								
**Total (NS, SC, FS, Non-FS)**	352	360	350	335	337	352	336	367
**Within linkage region**								
**Chr. 1: 181,329,185-211,652,186**	3	5	5	6	4	5	8	11
**Chr. 14: 36,888,531-58,397,917**	0	0	0	0	0	0	0	0
**Shared by all AFFECTED**	1 (*HMCN1*)	1	1	1	-	-	-	-

Exonic and splicing site variants were filtered based on being single nucleotide variants (SNVs) including non-synonymous (NS) and stop codon (SC) variants, while insertion/deletion (INDELs) including frame-shift (FS) and non-frame-shift (non-FS) variants. Novel variants were those not reported in dbSNP database (dbSNP 137) and rare variants (up to 2%) filter is based on the Japanese allele frequencies data from 1000 Genomes database. *Those family members were set as UNCONFIRMED because their affection status was based on clinical examination of the spleen.

Of the identified variants on chromosome 1, one was novel and the other was a rare variant. The novel variant (c.G361A, p.V121I) is located in the Contactin 2 gene (*CNTN2*) and it causes an amino acid substitution at exon 4. Similarly, the rare non-synonymous variant (rs150188026, c.G15614A, p.R5205H, MAF 0.018) is located in the Hemicentin 1 gene (*HMCN1*) and it causes an amino acid substitution at exon 101. Exome analysis of the Kosovo samples, revealed neither novel nor rare variants in *CNTN2*, but three of the four samples harbor very rare missense variants in *HMCN1* (c.C14111T, p.A4704V, MAF 0.001; c.C15011T, p.T5004I, MAF 0.008, and c.C15732G, p.H5244Q, MAF 0.005) (Table 2 and Table 3). The other identified novel variant of chromosome 14 is located to the DDHD domain containing 1 gene (*DDHD1*) (c.T1178C, p.V393A) and it causes an amino acid substitution at exon 4. However, examining Kosovo samples for variants in *DDHD1* did not reveal any novel or rare variants as defined by the previous criteria. Considering DDHD1 is an enzyme protein, it was possible to evaluate the variant’s impact by measuring the enzymatic activity. Functional impact of *DDHD1* variant (V393A) was evaluated by measuring activity of phospholipase A1 using phosphatidic acid as substrate, and no significant effect on the enzymatic activity of the variant protein was detected (data not shown).

**Table 2:** **Number of variants obtained using different filters in subjects affected by splenic epidermoid cyst from Kosovo**

**Criteria for variants filtering**	**Kos-1**	**Kos-2**	**Kos-3**	**Kos-4**
**Total (Exonic, Splicing)**	14817	14427	14783	14277
**SNVs (NS, SC)**	6701	6562	6734	6526
**Not in dbSNP or 1000 Genomes (novel)**	239	191	245	258
**Up to 2% in 1000 Genomes**	368	378	358	323
**INDELs (FS, non-FS)**	29	47	42	58
**Not in dbSNP or 1000 Genomes (novel)**	27	44	40	58
**Up to 2% in 1000 Genomes**	0	0	0	0

Exonic and splicing site variants were filtered based on being single nucleotide variants (SNVs) including non-synonymous (NS) and stop codon (SC) variants, while insertion/deletion (INDELs) including frame-shift (FS) and non-frame-shift (non-FS) variants. Novel variants were those not reported in dbSNP database (dbSNP 137) and rare variants filter is based on the European allele frequencies data from 1000 Genomes database.

**Table 3:** ***HMCN1*** **identified variants and scores of GERP and pathogenicity prediction tools**

**Substitution**	**Variant ID**	**Sample**	**MAF**	**GERP**	**SIFT**	**PolyPhen-2**
**R5205H**	rs150188026	II:2, II:6, III:2, IV:3, IV:4, IV:6	0.018	2.02	0.34	0.019
**T5004I**	rs114629728	Kos-2	0.005-0.011	2.56	0.29	0.175
**H5244Q**	rs75161007	Kos-3	0.005-0.022	0.69	0.60	0.010
**A4704V**	rs41317503	Kos-4	0.001	5.47	0.04	0.774

*Abbreviations: MAF* Minor allele frequency (based on 1000 Genomes allele frequencies for Japanese and European populations), *GERP* Genomic evolutionary rate profiling.

### Sanger sequencing confirmation and co-segregation

We also performed Sanger sequencing to confirm the three candidate variants obtained from exome sequencing for all of the family members and Kosovo patients. Both V121I variant of *CNTN2* and R5205H of *HMCN1* showed consistent co-segregation with the affection status except III:3, III:4, and IV:1, because a haplotype harboring the two variants is co-segregating without recombination in the family (Figure 2). The incompleteness of co-segregation in III:3 who is an unaffected male with heterozygote genotype but also the father of the affected 14 years old child (IV:6). Inconsistency of co-segregation in III:3 and III:4 family members may be due to the incomplete penetrance of the disease. The variant V393A of *DDHD1* is less consistently co-segregated with II:3, II:5, III:3, and III:5 having a heterozygote genotype while they are unaffected (see Additional file 2). We further focused on *HMCN1* because it showed a better co-segregation with the disease and other variants were detected in Kosovo samples therefore it seemed a good candidate for disease causality.

### *HMCN1* variants’ genomic context, conservation, and pathogenicity

**Figure 4:**
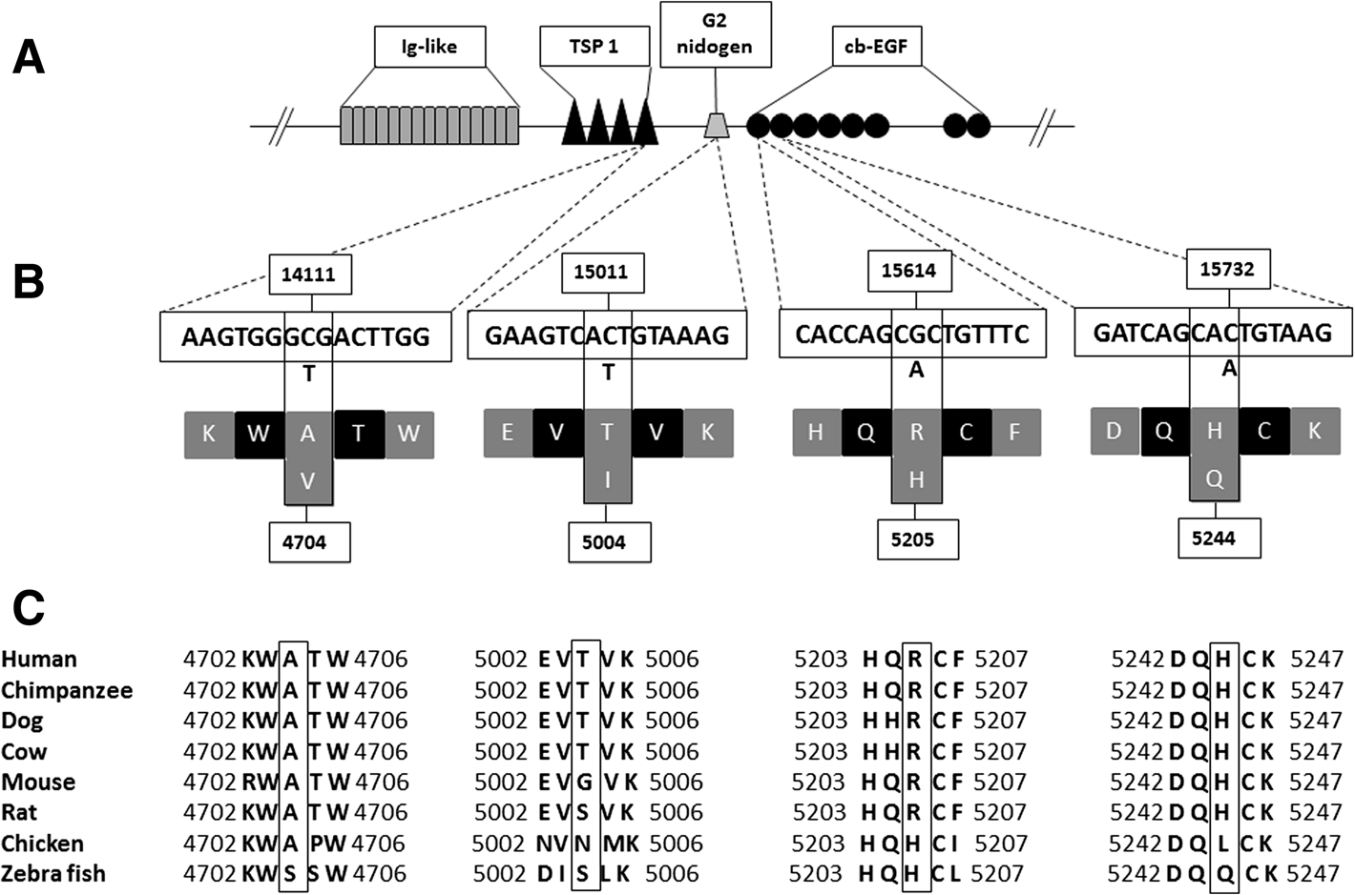
**Genomic context of** ***HMCN1*** **domains and variants with their across-species conservation. (A)** Domain structures of partial *HMCN1* are illustrated. TSP1 denotes thrombospondin type 1 domain and cb-EGF denotes calcium-binding epidermal growth factor like domain. The repetitive Immunoglobulin like (Ig-like) domain is also presented. **(B)** The genomic context of *HMCN1* variants and the corresponding amino acid substitutions with their physical position. **(C)** Across-species conservation of the variants’ positions indicating a conserved pattern except for chicken and zebrafish.

Using InterPro database to predict the genomic context of the variants, all of the identified *HMCN1* variants are located within conserved domains [26]. Two of the variants, R5205H and H5244Q, are located on the calcium-binding Epidermal Growth Factor (cb-EGF) like domain, while the variant, A4704V, is located on the Thrombospondin type 1 (TSP1) domain and the variant, T5004I, is on the G2 nidogen domain (Figure 4). Sequence homology of the variants’ vicinities between several species showed a relatively high conservation among mammalian species (Figure 4). Furthermore, annotation of variants’ across-species conservation, using Genomic Evolutionary Rate Profiling (GERP) score as a measurement, revealed that most of the variants have relatively moderate conservation scores, which ranges from 0.69 to 5.47 (Table 3). We also used the pathogenicity prediction tools SIFT [27] and PolyPhen2 [28] to predict the impact of *HMCN1* variants on the protein activity (Table 3).

## Discussion

Splenic epidermoid cyst is a very rare disease and we recruited a three-generation Japanese family comprising six patients. In the study, we first performed genome-wide parametric linkage analysis to narrow down the candidates loci. We then performed exome re-sequencing for eight individuals from the family in addition to four unrelated affected individuals from Kosovo. Based on stringent filtering steps on the exome data, in combination with linkage results, three variants of *CNTN2*, *HMCN1,* and *DDHD1* were initially identified in the family. Because we could not pinpoint to one gene, it was necessary to find additional evidence supporting their possible role in the disease. We considered if any of the Kosovo unrelated samples has a novel or rare variant in the same gene that would prioritize the gene as candidate. Interestingly, we found rare missense variants in only *HMCN1* in both the affected Japanese and three Kosovo cases.

HMCN1 (also known as Fibulin 6) is a large extracellular member of the immunoglobulin superfamily and it contains around 5,635 amino acids (600 kD) [29] [UniProtKB**:**Q96RW7]. This protein belongs to a family known as fibulins, a group of related proteins involved in many cellular processes such as cell adhesion, migration, and proliferation [30]. Expression of *HMCN1* mRNA in the human spleen is relatively higher than other organs [31], and HMCN1 protein was detectable in the pericellular extracellular matrix of mouse epithelial cells in many tissues [32]. Function of human HMCN1 is still not well investigated, however a similar protein in *C. elegans* forms fine tracks that are mainly involved in stabilizing and organizing extracellular matrix of the epidermis [29]. Variants of *HMCN1* have been reported to associate with age-related macular degeneration, a phenotype that is characterized by extracellular deposits of proteins and lipids in the eye [33]. Studies from zebrafish also revealed that variants of *Hmcn1* caused a phenotype named fin blistering [34], which is a fluid-filled structure encompassed by a layer of epithelial cells. Considering that splenic epidermoid cyst has a structure filled with fluids and lined by a layer of epithelial cells, these phenotypic similarities with the previous observations would support the possible involvement of *HMCN1* in the etiology of splenic epidermoid cyst.

The molecular mechanism by which *HMCN1* is likely involved in splenic epidermoid cyst still remains unknown. However, one scenario that might explain the implication of *HMCN1* is related to its function as an extracellular matrix protein. As noted previously, one of the hypotheses of splenic cyst formation is that a misplacement of epithelial cells during developmental stages. Bearing that in mind, it is possible that such misplacement could be due to instability of HMCN1 structure caused by the variants of the gene. Another interesting idea is that the domain cb-EGF, which is mutated in our study subjects, requires Ca^2+^ ion for orienting the neighboring domains and inducing protein conformations required for biological activities [35]. Interestingly, Ca^2+^ ion homoeostasis is known to be a major factor in cyst formation in polycystic kidney diseases [36].

Our study has a limitation related to the number of subjects involved; because it includes only one family with the disease, which is basically due to the rarity of the disease. However, we believe that is compensated by the detection of variants on the same gene from a completely different population such as Kosovo population. Further supporting evidences are warranted to support our hypothesis. Firstly, more splenic cyst patients need to be screened for variants in *HMCN1*. Secondly, functional impact of *HMCN1* variants on epidermoid transformation and splenic cyst formation needs to be experimentally shown. For example, localization of HMCN1 protein in the cyst’s samples would be shown with immunohistochemical method. One more example is using a forward genetic approach to knock out *Hmcn1* from mouse and if such experiment showed a phenotype similar to splenic cyst, this will clearly enforce the hypothesis of *HMCN1* involvement in splenic epidermoid cyst etiology.

## Conclusions

In the current study, taking the advantage of linkage and exome analyses, we demonstrated that variants of *HMCN1* are strong candidates for the causality of splenic epidermoid cyst. To our knowledge, this is the first study to investigate the genetics of splenic epidermoid cyst identifying *HMCN1* as the causal gene.

## Additional files

Additional file 1:
**Genetic distances and LOD scores in a data set used for linkage analysis.** The file is an example data set of the ones used in linkage analysis and it includes markers, genetic distances and LOD score values. Additional file 2:
**Pedigree of the studied Japanese family with the genotypes of V393A of** ***DDHD1.*** Filled squares and circles denote affected individuals and open symbols represent unaffected subjects. The arrow indicates the proband (IV:3). The genotypes are for the V393A variant of *DDHD1* and they show an incomplete co-segregation with the affection status.
