# Supplementary figures and images for: Genome-wide linkage and exome analyses identify variants of HMCN1 for splenic epidermoid cyst
Source: BMC Med Genet. 2014 Oct 23;15:115. doi: 10.1186/s12881-014-0115-4 (PMC4258954; doi:10.1186/s12881-014-0115-4)

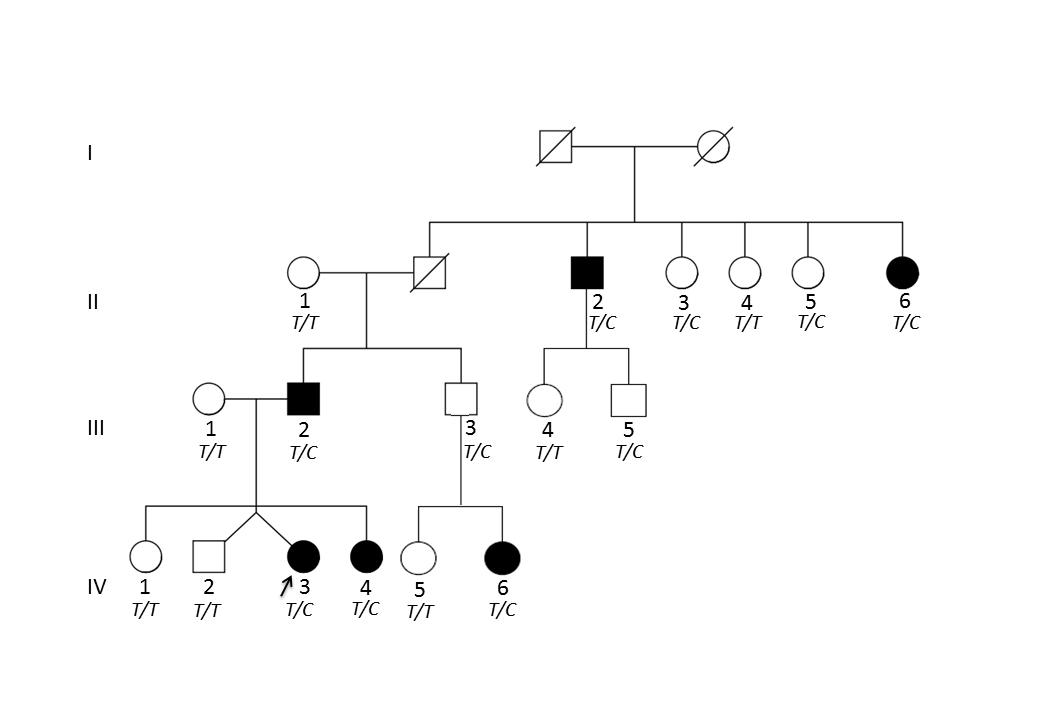

Supplement: Additional file 2: — Pedigree of the studied Japanese family with the genotypes of V393A of DDHD1. Filled squares and circles denote affected individuals and open symbols represent unaffected subjects. The arrow indicates the proband (IV:3). The genotypes are for the V393A variant of DDHD1 and they show an incomplete co-segregation with the affection status. [file 12881_2014_115_MOESM2_ESM.png]
